# Hyperoside induces cell cycle arrest and suppresses tumorigenesis in bladder cancer through the interaction of EGFR-Ras and Fas signaling pathways

**DOI:** 10.7150/ijms.90261

**Published:** 2024-02-12

**Authors:** Kai Yang, Zhi-Xiang Qi, Ming-Xin Sun, Li-Ping Xie

**Affiliations:** Department of Urology, the First Affiliated Hospital, Zhejiang University School of Medicine, Hangzhou, Zhejiang 310003, P.R. China.

**Keywords:** bladder cancer, hyperoside, cell cycle arrest, Ras, Fas, MAPK

## Abstract

Hyperoside is a natural flavonol glycoside widely found in plants and has been reported to have a variety of pharmacological effects, including anticancer abilities. In this study, we demonstrated for the first time that hyperoside inhibited the proliferation of bladder cancer cells in vitro and in vivo. Moreover, hyperoside could not only induce cell cycle arrest, but also induce apoptosis of a few bladder cancer cells. Quantitative proteomics, bioinformatics analysis and Western blotting confirmed that hyperoside induced the overexpression of EGFR, Ras and Fas proteins, which affects a variety of synergistic and antagonistic downstream signaling pathways, including MAPKs and Akt, ultimately contributing to its anticancer effects in bladder cancer cells. This study reveals that hyperoside could be a promising therapeutic strategy for the prevention of bladder cancer.

## Introduction

Bladder cancer is the fourth most common cancer in men in the United States. In fact, there were an estimated 81,180 new cases and 17,100 deaths from bladder cancer in the United States in 2022 1. In addition, 573,278 new cases of bladder cancer and 212,536 bladder cancer deaths were predicted to occur globally in 2020 2. Although chemotherapy has revolutionized the treatment of advanced tumors 3, the median survival of cisplatin combined chemotherapy for metastatic disease can only be extended to 14 months 4, and the associated side effects due to the lack of specificity to tumor cells remain a challenging issue. Therefore, there is an urgent need for new therapeutic strategies to treat advanced bladder cancer.

The incidence of bladder cancer varies across countries and regions, with the highest rates in Southern Europe (Greece, Spain, and Italy), Western Europe (Belgium and the Netherlands), and Northern America 2. However, the regions with the lowest incidence, such as Western and Central Africa and Southeastern Asia, have rates five to ten times lower than those in the United States and Europe 2. Due to geographic discrepancies in incidence, more and more scholars have begun to pay attention to the effects of diet, nutritional intake, and some chemopreventive drugs on tumorigenesis.

Flavonoids, a class of polyphenols, are common bioactive secondary metabolites and components in vegetables and fruits 5, and hold great promise in cancer chemoprevention and chemotherapy due to their high availability, strong anticancer efficiency and low toxicity 6. Hyperoside (quercetin-3-O-galactoside) is a natural flavonol glycoside widely existing in Hypericaceae, Rosaceae, and other plants 7, 8, and has various pharmacological effects such as anticancer, anti-inflammatory, antioxidant, organ protection, antibacterial, antiviral, etc. 9, 10.

However, the antitumor effect of hyperoside on bladder cancer has not been studied, and its exact mechanism and targets remain unclear 9, 10. In this study, we reported for the first time that hyperoside induced cell cycle arrest and a small amount of apoptosis in bladder cancer cells, and inhibited tumor growth in xenografted tumor models in nude mice.

## Materials and Methods

### Reagents and antibodies

Hyperoside (>98% pure) was obtained from Tanmo Quality Control Technology Co. (Changzhou, China) and 3-(4,5-dimethylthiazol-2-yl)-2,5-diphenyltetrazolium bromide (MTT) was purchased from Sigma Chemical Co. (St. Louis, MO, USA). The annexin V-FITC apoptosis detection kit was purchased from BD Biosciences (San Jose, CA, USA). The bicinchoninic acid protein assay kit was purchased from Pierce Biotechnology (Rockford, IL, USA). The Coulter DNA Prep™ Reagents Kit was purchased from Beckman Coulter (Fullerton, CA, USA). Antibodies were purchased from Cell Signaling Technology (Beverly, MA, USA) and Santa Cruz Biotechnology (Santa Cruz, CA, USA).

### Cell culture

Human bladder cancer cell lines T24 and 5637 were derived from the Shanghai Institute of Cell Biology, Chinese Academy of Sciences. The cells were cultured in RPMI 1640 medium (HyClone, Logan, UT, USA) supplemented with 10% heat-inactivated fetal bovine serum, penicillin (100 U/mL) and streptomycin (100 mg/L) in a humidified atmosphere containing 5% CO_2_ at 37°C. The day before treatment, the cells were inoculated in an antibiotic-free growth medium at a density of 30-40%. Cell images were taken using a phase contrast microscope (Olympus, Japan) with 100× magnification.

### Cell growth/viability assay

The cell proliferation was measured by MTT assay. Approximately 3000 to 8000 cells (depending on their culture time) were plated in each well of the 96-well plate. After incubation overnight, cells were treated with different concentrations of hyperoside (0-800 μM) for 24 h. The medium was removed at different times after treatment, and plates were incubated at 37°C for 4 hours after adding 20 μl MTT (5 mg/ml) into each well. After that, the purple formazan precipitated in each well was dissolved in 150 μl dimethyl sulfoxide while the plates were spun in a centrifuge. Measurement of absorbance at 490 nm was performed on an absorbance reader (MRX II; DYNEX Technologies, Chantilly, VA, USA). The reduction in cell viability in each group was expressed as a percentage of control cells, which were considered to be 100% viable.

### Cell cycle analysis by flow cytometry

Cell cycle analysis was performed using the Coulter DNA Prep™ Reagents Kit. Cells were plated in six-well plates and incubated overnight before treatment. After treatment for 24h, harvested cells were washed twice with prechilled PBS and resuspended with 100 μl PBS at a concentration of 1×10^6^ cells/ml. Each cell sample was mixed with 100 μl DNA Prep LPR, gently mixed with a vortex, and incubated in the dark at room temperature (25°C) for 20 minutes. Then, each sample was mixed with 1 ml of DNA Prep Stain, mixed gently with a vortex, and incubated again in the dark at room temperature (25°C) for 20 minutes. Finally, the flow cytometry system (Beckman Coulter FC500 Flow Cytometry System with CXP Software; Beckman Coulter, Fullerton, CA, USA) was used for cell cycle analysis within 1 hour, and raw data was analyzed by Multicycle for Windows (Beckman Coulter).

### Detection of apoptotic cells by flow cytometry

Apoptosis was quantitatively assessed by determining the percentage of cells with highly condensed or fragmented nuclei. The cells were plated in six-well plates and incubated overnight before treatment. As mentioned above, the cells were harvested 24 hours after hyperoside treatment for 24 hours, washed twice with prechilled PBS, and resuspended with 100 μl 1× binding buffer at a concentration of 1×10^6^ cells/ml. Double staining was performed with fluorescein isothiocyanate (FITC) conjugated annexin V and propidium iodide (PI) (Annexin V-FITC Apoptosis Detection Kit) according to the manufacturer's protocol. The apoptosis was analyzed by flow cytometry within 1 hour.

### Western blotting analysis

Briefly, cells were harvested 24 hours after hyperoside treatment as described above, washed and lysed with a lysis buffer. The protein concentration in the lysate was determined using a bicinchoninic acid protein assay kit. An appropriate amount of protein (30-50 µg) was isolated by electrophoresis in 10-15% Tris-glycine polyacrylamide gel and transferred to nitrocellulose membrane. The membrane was blocked and then incubated overnight with the appropriate primary antibody at a dilution specified by the manufacturer. The membrane was then washed three times in 15 ml TBST and incubated with the corresponding horseradish peroxidase (HRP) conjugated secondary antibody at a 1:1000 dilution in TBST for 1 hour. The combined secondary antibody was detected using an enhanced chemiluminescence (ECL) system (Pierce Biotechnology Inc., Rockford, IL, USA) after three washes with 15ml TBST for 5 minutes each.

### Mass spectrometry and bioinformatics analyses

In brief, T24 cells were treated with 400 μM hyperoside for 24 h and then homogenized in SDT buffer (4% SDS, 100 mM DTT, 150 mM Tris-HCl pH 8.0). The supernatant was quantified with BCA Protein Assay Kit (P0012, Beyotime). After further digestion by trypsin using filter-assisted sample preparation, the peptide content was estimated by UV spectral density at 280 nm using an extinction coefficient of 1.1 of 0.1% (g/l) solution. A 100 μg peptide mixture of each sample was labeled using TMT reagent according to the manufacturer's instructions (Thermo Fisher Scientific) and fractionated by reverse-phase chromatography using Agilent 1260 infinity II high performance liquid chromatography (HPLC). Then, each fraction was injected for nano LC-MS/MS analysis. LC-MS/MS analysis was performed on a Q Exactive Plus mass spectrometer (Thermo Fisher Scientific), which was coupled to the Easy nLC (Thermo Fisher Scientific) for 60/90 min. MS/MS raw files were processed using the MASCOT engine (Matrix Science, London, UK; version 2.6) embedded into Proteome Discoverer 2.2. A 1% false discovery rate (FDR) was set to identify proteins. The Gene Ontology (GO) annotation was completed by Blast2GO Command Line (www.geneontology.org). Pathway enrichment analysis was performed using the KEGG (Kyoto Encyclopedia of Genes and Genomes) database (http://www.kegg.jp/). Protein-protein interaction prediction was conducted using the STRING Version 11.0 (https://string-db.org/).

### Animal experiments

Male BALB/c-nude mice weighing 18-20 g each (4 weeks old, provided by the Shanghai Experimental Animal Center, Chinese Academy of Sciences, Shanghai, China) were used for the experiments. The mice ate and drank freely and were fed in a 12:12 h light:dark cycle with room temperature maintained at 21℃. T24 bladder cancer cells (1×10^5^ in 10 μL PBS) were injected s.c. into the right flank of mice. The growth rate of tumors was monitored and measured by a blinded observer. The formula for calculating tumor volume is: volume (mm^3^) = width^2^ (mm^2^) × length (mm) × 0.52, where length and width were tumor diameters measured with calipers in mutually perpendicular directions. When the tumor size reached about 30 mm^3^, the mice were divided into three groups and treated with an i.p. injection of propylene glycol (vehicle, 0.1 mL), 20 mg/kg hyperoside (in 0.1 mL propylene glycol), or 40 mg/kg hyperoside (in 0.1 mL propylene glycol) once daily for 3 weeks. Tumor size was measured every two days for 21 days.

### Statistical analysis

All in vitro experiments were performed in triplicate. GraphPad Prism software (San Diego, CA, USA) was used to calculate statistically significant differences with Student's *t* test. All values are expressed as means ± SDs, and P < 0.05 was considered statistically significant.

## Results

### Hyperoside inhibits cell growth in bladder cancer cells

**Figure 1:**
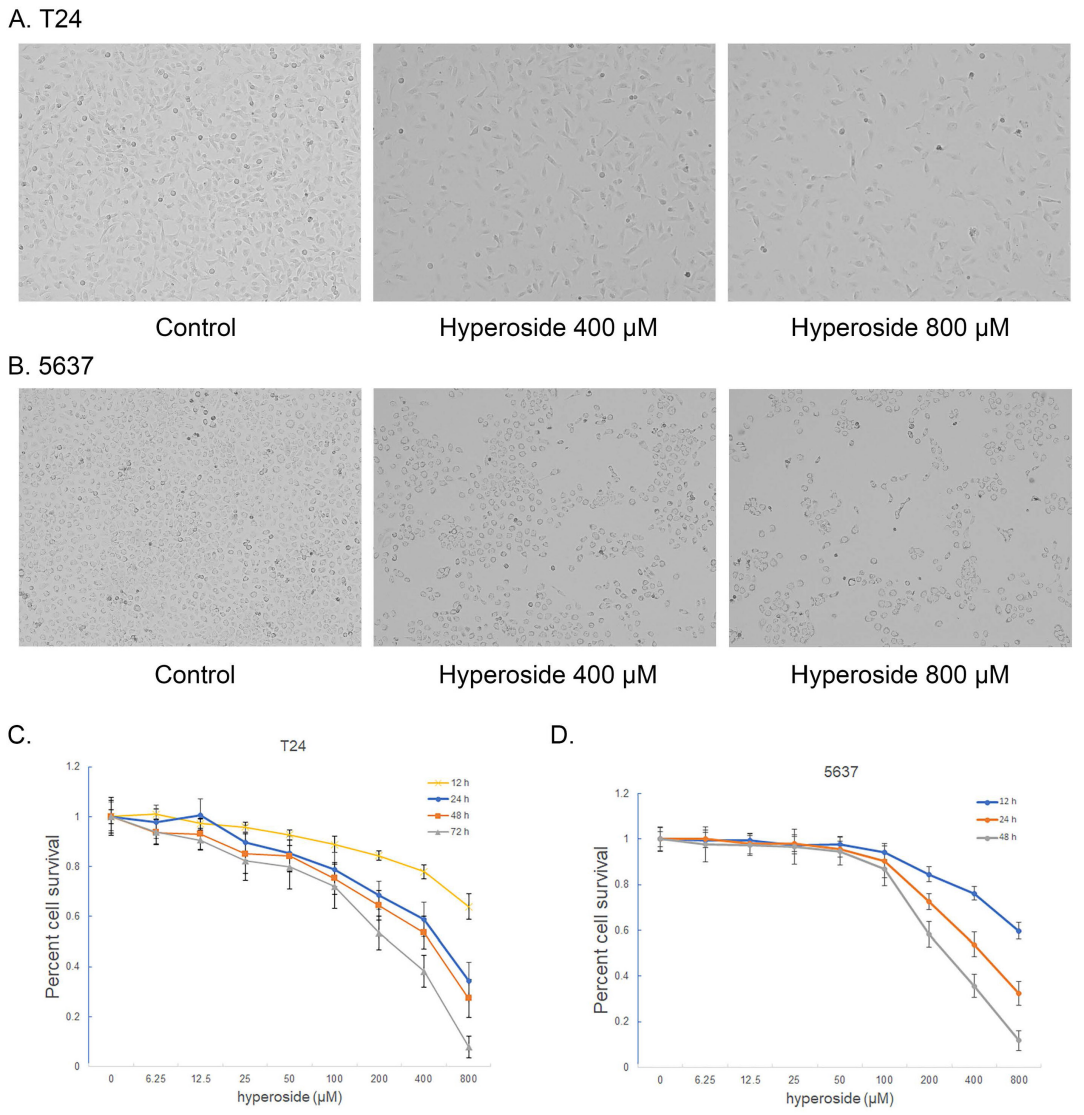
Hyperoside induces growth inhibition of T24 (a) and 5637 (b) bladder cancer cells. Cells were treated with 0, 400 or 800 μM hyperoside. Cell images were taken at 48 h after transfection at 100× magnification. Hyperoside-treated cells were less dense and had more dead cells than controls. Hyperoside inhibited the viability of T24 (c) and 5637 (d) cells in a dose-dependent and time-dependent manner, as assessed by the MTT assay. Reduced cell viability was observed after hyperoside treatment (0 to 800 μM) at 24, 48, and 72 hours. Data are presented as the means ± SDs (n = 8).

To investigate the effects of hyperoside on human bladder cancer cells, T24 and 5637 cells were treated with 400 or 800 μM hyperoside for 12 to 48 hours and found to gradually lose their viability and stop proliferating (Fig. 1a and 1b). The cytotoxic effects of hyperoside on T24 and 5637 cells at varying concentrations and times (12 - 72 h) were determined by MTT assay. As shown in Fig. 1, the effects of hyperoside on cell viability, which were dose and time dependent, occurred within 12 h and at concentrations as low as 25 μM. Compared with the control cells treated with 0.1% DMSO, the viability in T24 cells decreased by 4.2% to 36.0% after treatment with 25 - 800 μM hyperoside for 12 h, whereas it deceased by 10.4% to 65.8%, 14.8% to 72.7% and 17.7% to 92.2% after 24, 48 and 72 h, respectively (Fig. 1c). Similarly, the reduction of viability in 5637 cells ranged from 2.7% to 40.2%, 1.8% to 67.5% and 3.3% to 88.2% after hyperoside treatment for 12, 24 and 48 h, respectively (Fig. 1d). The inhibitory concentration 50% (IC50) of hyperoside treatment on T24 cells were about 629, 330, 252 and 159 μM at 12, 24, 48 and 72 h, respectively. Moreover, the IC50 values of hyperoside in 5637 cells were approximately 667, 431 and 250 μM at 12, 24 and 48 h, respectively.

### Hyperoside induces cell cycle arrest in bladder cancer cells

**Figure 2:**
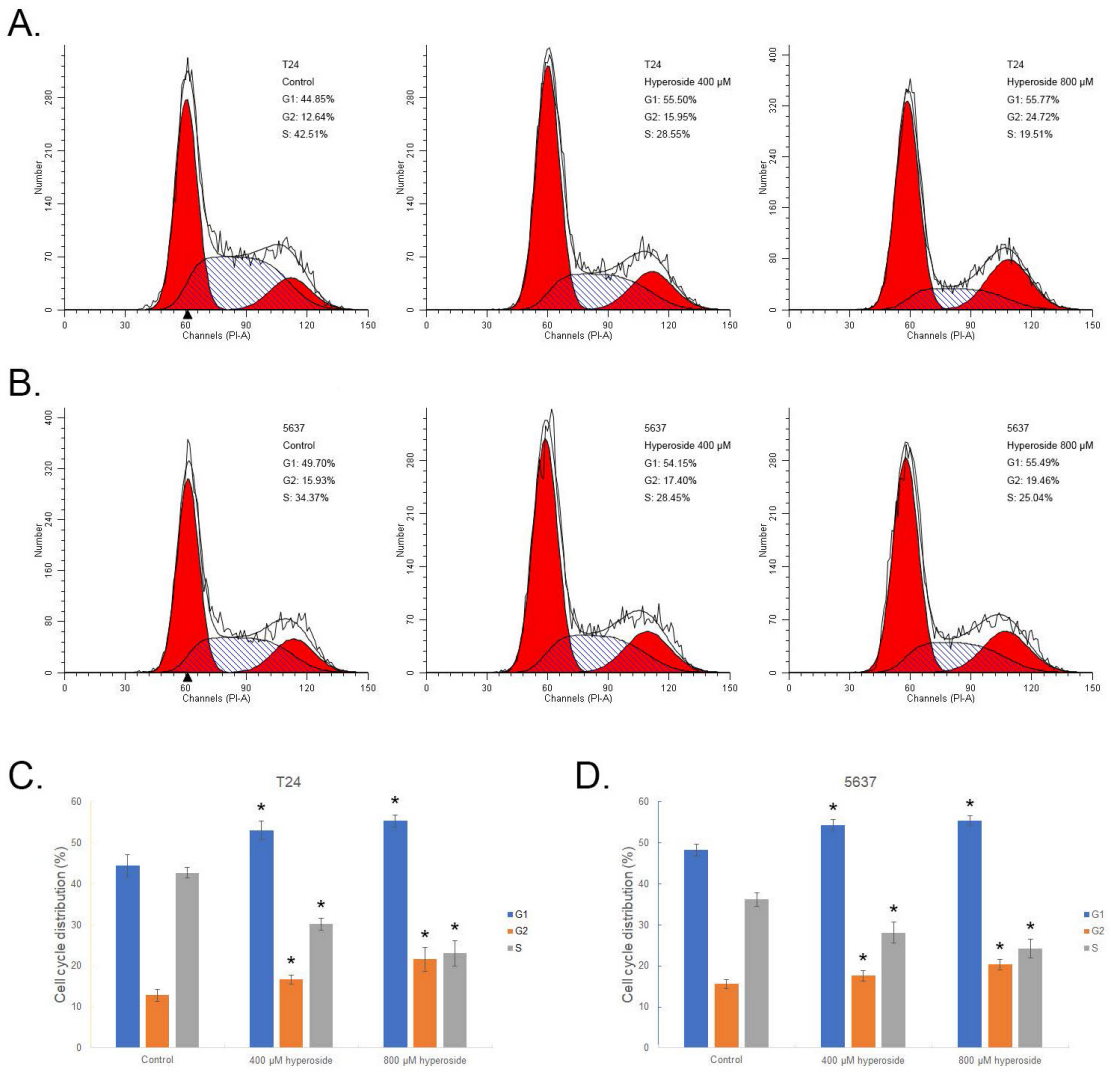
Hyperoside induces cell cycle arrest in T24 (a) and 5637 (b) bladder cancer cells, as detected by flow cytometry. Cells were treated with 400 - 800 μM hyperoside for 48 hours. The sub-G0/G1 cells were not included in the calculations. Representative images from three independent experiments with identical results are shown. (c, d) Flow cytometry data were analyzed to compare cell cycle distribution (means ± SDs from three independent experiments). The percentages of G1-, G2- and S-phase cells are shown. * p < 0.05.

Flow cytometry was used to study the relationship between hyperoside-mediated inhibition of cell proliferation and cell cycle arrest. As shown in Fig. 2a, G1 and G2 phase arrest was observed in 400 - 800 μM hyperoside-treated T24 cells. The number of G1 phase cells treated with 400 and 800 μM hyperoside was approximately 55% at 48 h following treatment and increased by more than 10% compared to the control group. In addition, the number of G2 phase cells treated with 400 - 800 μM hyperoside also increased by 3% - 12%. The increase in the G1 and G2 phase cells was found to be associated with a concomitant significant decrease in the S phase population (Fig. 2a and 2c). A similar phenomenon of G1 and G2 phase cell cycle arrest was also observed in 5637 bladder cancer cells (Fig. 2b and 2d).

**Figure 3:**
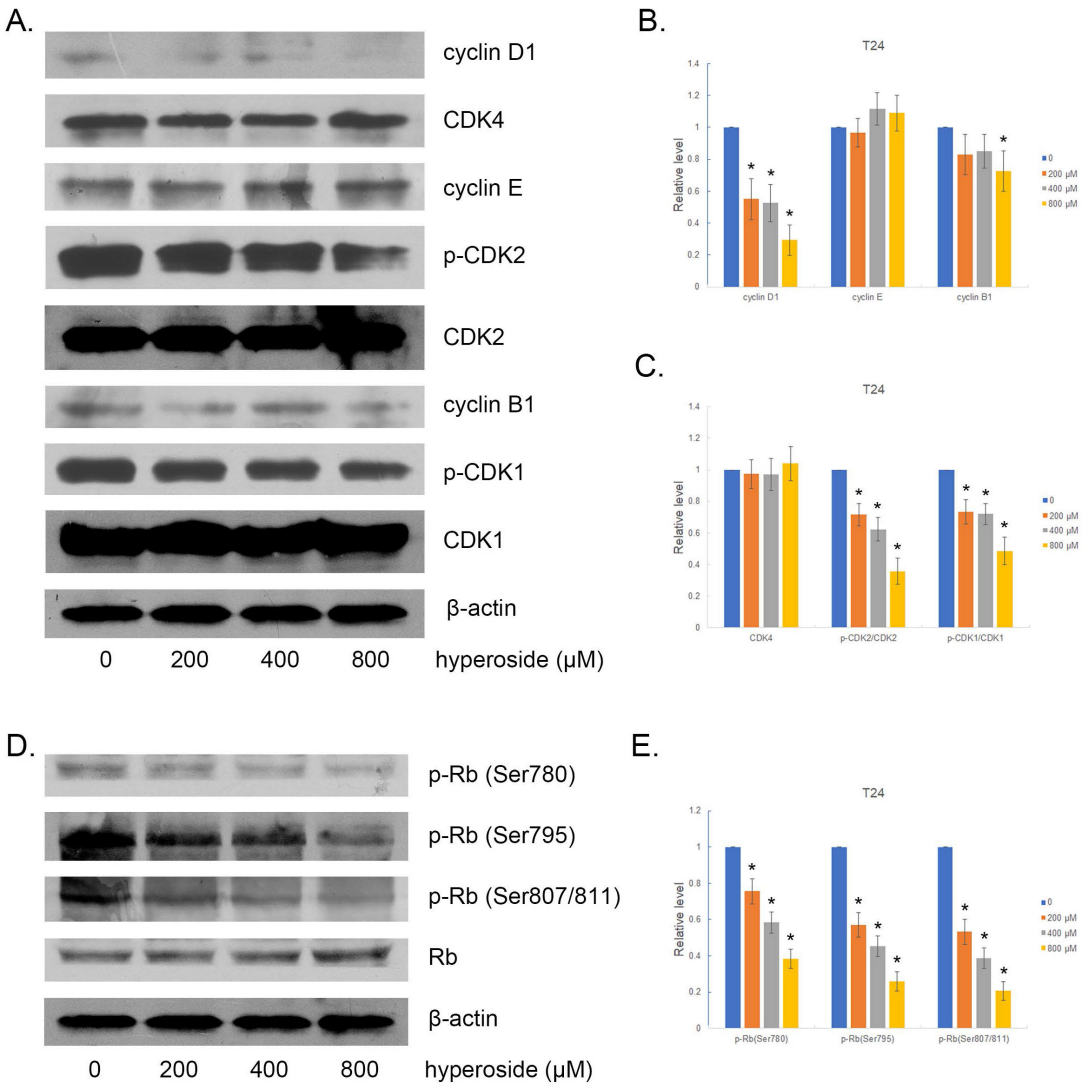
The effects of hyperoside on cell cycle-related proteins in T24 bladder cancer cells. Cells were treated with 200 - 800 μM hyperoside for 48 hours. (a, d) Representative Western blot images of cyclins, CDKs, and Rb from three independent experiments with identical results are shown. (b, c, e) The expression of cyclins, CDKs, and Rb was analyzed. Values are mean ± SD. * p < 0.05.

We then examined the expression of several cell cycle-related proteins. Accordingly, interference with the cell cycle was associated with decreased expression of cyclin D1 and reduced phosphorylation of CDK2 and CDK1 in hyperoside-treated T24 cells. We also found a small decrease in cyclin B1 expression in hyperoside-treated cells, but no alteration in cyclin E and CDK4 expression (Fig. 3a-3c). As a result, the phosphorylation level of Rb protein, a key point in the cell cycle to propel cell proliferation 11, is significantly reduced (Fig. 3d and 3e).

### Hyperoside induces apoptosis in a small number of bladder cancer cells

**Figure 4:**
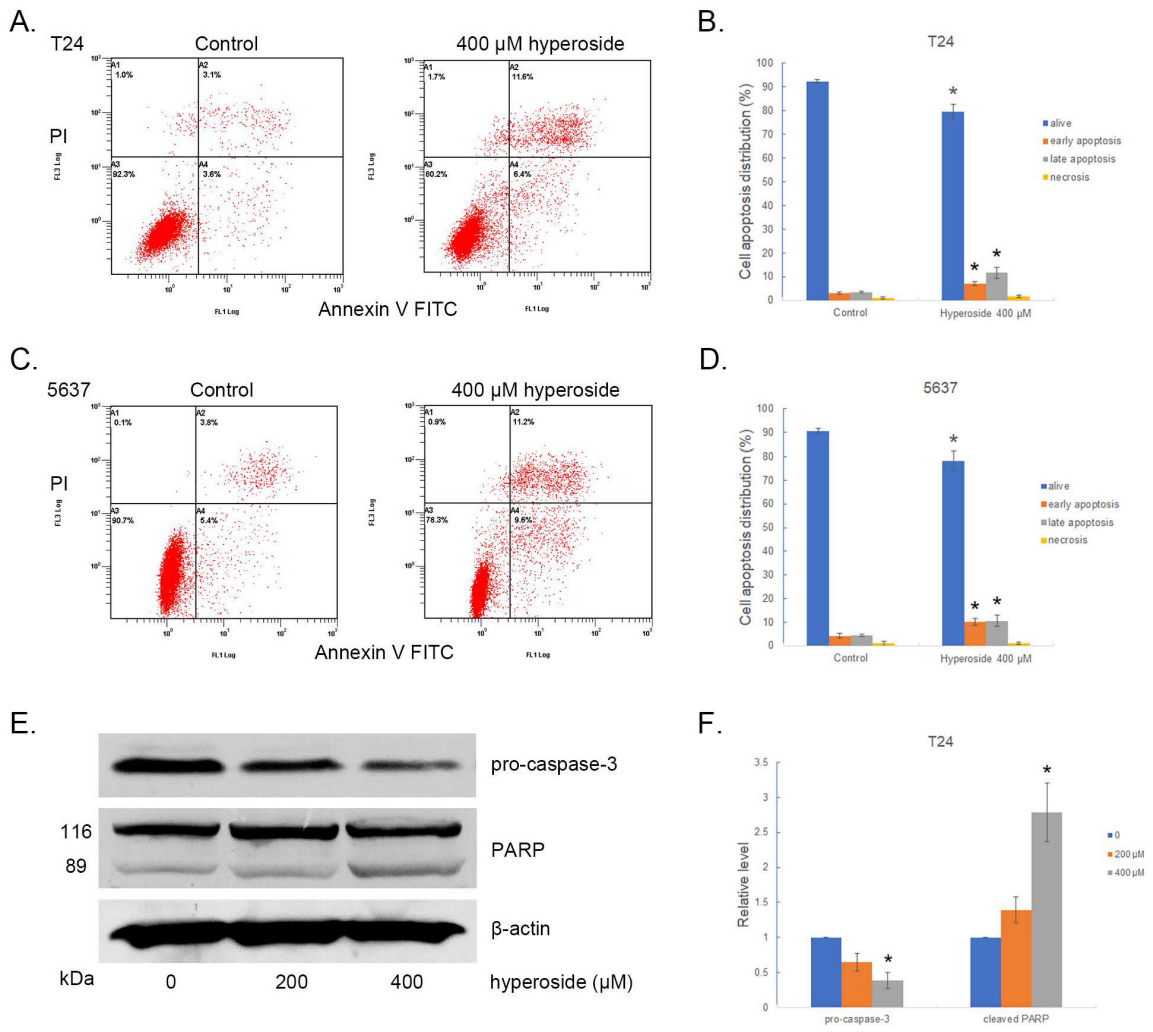
Hyperoside induces cell apoptosis in T24 (a) and 5637 (c) bladder cancer cells. Cells were treated with 400 - 800 μM hyperoside for 48 hours and detected by flow cytometry using a double-staining method with fluorescein thiocyanate-conjugated annexin V and propidium iodide. Annexin V-stained cells indicate early apoptotic cells, whereas Annexin V + propidium iodide-stained cells are late apoptotic cells. (b, d) Flow cytometry data were analyzed to compare cell apoptosis populations (means ± SDs from three independent experiments). The percentages of live, apoptotic and necrotic cells are shown. (e) Hyperoside treatment activated caspase-3 and poly (ADP-ribose) polymerase (PARP) in T24 cells. (f) The expression of pro-caspase-3 and PARP was analyzed. Representative images and blots from three independent experiments with identical results are shown. Values are mean ± SD. * p < 0.05.

We next analyzed the relationship between hyperoside-mediated loss of cell viability and apoptosis by flow cytometry, and found that hyperoside induced mild apoptosis in bladder cancer cells T24 and 5637 after 400 μM hyperoside treatment for 48 h. As shown in Fig. 4a-4d, the proportion of late apoptotic cells (UR quadrant) increased to more than 10%, and the early apoptotic cells (LR quadrant) also increased slightly compared with the control group.

Caspase-3 and poly (ADP-ribose) polymerase (PARP) play a central role in apoptosis. Accordingly, significantly reduced levels of pro-caspase-3 were observed in T24 cells treated with 200 - 400 μM hyperoside for 48 h (Fig. 4e and 4f). Moreover, 89 kDa cleaved PARP fragments were detected in the hyperoside-treated samples. Thus, the evident changes in apoptosis-related proteins induced by hyperoside treatment confirmed the ongoing apoptosis and the antitumor effects on human bladder cancer cells.

### Quantitative proteomics and bioinformatics analyses suggest the involvement of EGFR-RAS and Fas signaling induced by hyperoside

**Figure 5:**
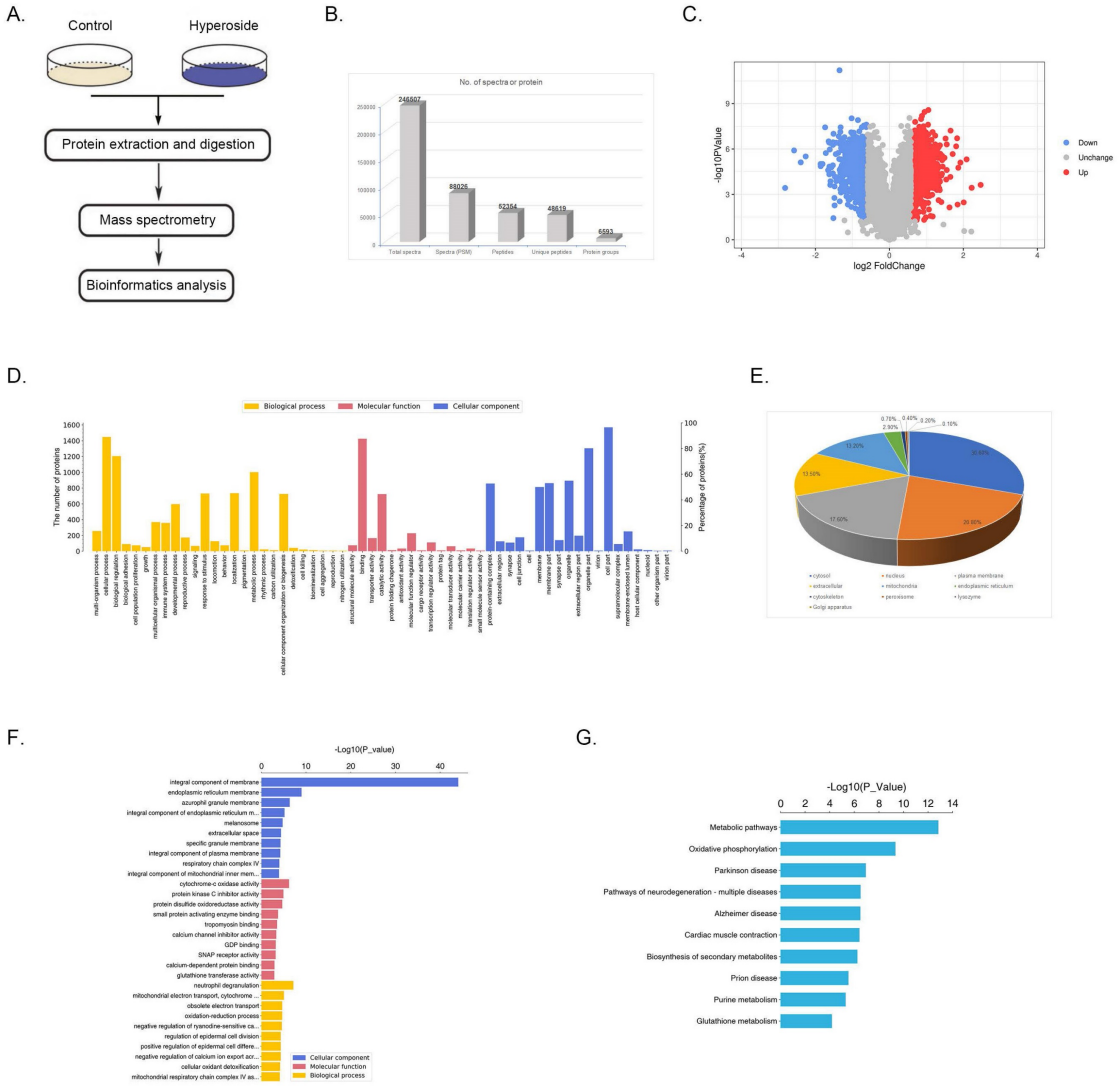
Quantitative proteomics and bioinformatics analysis of hyperoside treatment in T24 bladder cancer cells. (a) Technical schematic diagram of the identification of hyperoside-regulated proteins by mass spectrometry analysis. (b) Results of the LC-MS/MS for the proteins. (c) The volcano plot shows the up- (red) or downregulated (blue) proteins between the hyperoside treated groups and the controls. (d) Biological processes, molecular functions and cellular components of GO annotation were performed by Blast2GO. (e) WoLF PSORT-based subcellular localization prediction. (f) GO enrichment analysis of molecular function, cellular component and biological process ontologies. (g) KEGG pathway enrichment analysis of the dysregulated proteins.

To explore the potential mechanism responsible for hyperoside-induced cell cycle arrest and apoptosis, quantitative proteomics was used to detect the proteomic alterations in T24 cells treated with 400 μM hyperoside for 24 h. Total cell proteins were collected and digested with trypsin for LC-MS/MS proteomics analysis based on tandem mass tag (TMT) (Fig. 5a). As shown in Fig. 5b, 246,507 spectra were obtained from TMT-based proteomics analysis. After data filtering, the low-scoring spectra was eliminated and 88,026 spectra were matched to 52,354 peptides in the database, of which 48,619 were unique peptides. Finally, 6,593 proteins were identified. For comparison of hyperoside-treated cells with control groups, a protein was regarded as a differentially expressed protein (DEP) if the fold change is > 1.5 or < 0.67 and the P value is < 0.05. Based on these two criteria, 1,627 DEPs were identified, of which 874 were significantly upregulated and 753 were downregulated in hyperoside-treated T24 bladder cancer cells compared with controls (Fig. 5c). These DEPs were mainly clustered into 59 GO functional categories, involving 26 biological processes, 19 cellular components, and 14 molecular functions (Fig. 5d).

To further understand the function of these proteins, we used WoLF PSORT to predict the subcellular localization of these DEPs. The results indicated that the largest subcellular fraction was cytosol, accounting for 30.6% of the DEPs, and other significant subcellular location sites were nucleus (20.8%) and plasma membrane (17.6%) (Fig 5e). GO enrichment analysis is shown in Fig. 5f, and the most significantly enriched cellular components were integral components of the membrane. Then, KEGG analysis was conducted to study the enriched pathways related to the dysregulated proteins, and the most enriched pathways were found to be the metabolic pathways (Fig. 5g).

**Figure 6:**
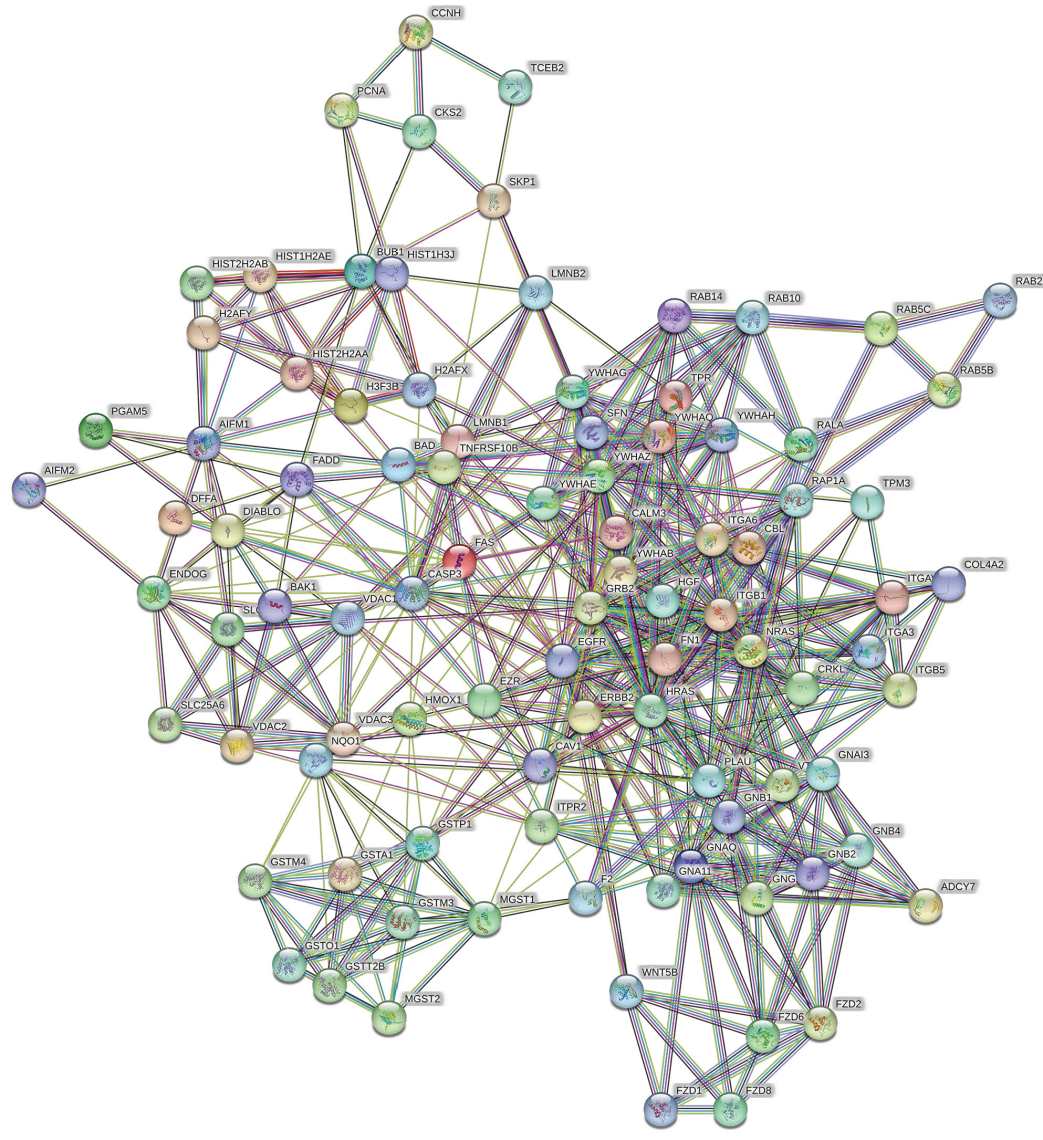
Protein-protein interaction network analysis was conducted with STRING Version 11.0 and suggested the potential importance of the EGFR-Ras and Fas signaling pathways.

To better understand the potential protein-protein interactions (PPIs) of the DEPs and the related unaltered proteins, we subsequently performed a PPI proteomics network analysis using STRING software. The interactions of robust and crosstalk signaling are mapped in Fig. 6. Network analysis of the differentially expressed proteins suggested that EGFR-Ras and Fas signaling pathways might play a key role in mediating the effects of hyperoside on bladder cancer cells.

### The alteration of Fas and EGFR-Ras related signaling pathways induced by hyperoside

**Figure 7:**
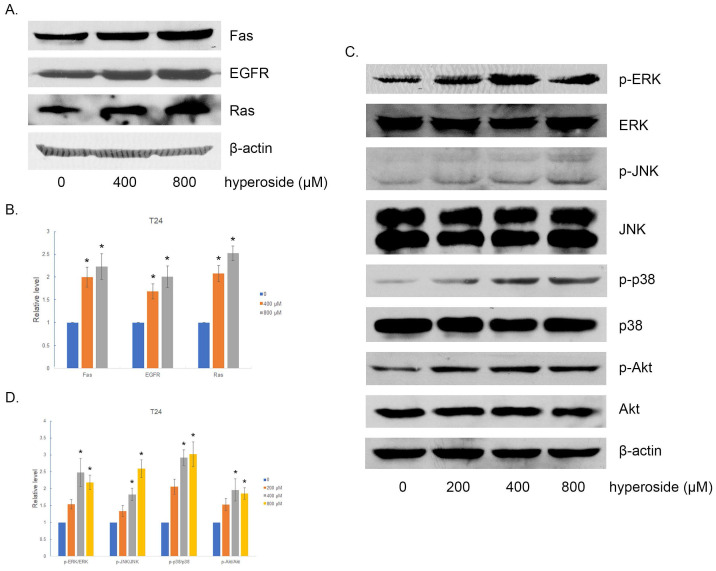
The effects of hyperoside on Fas, EGFR, Ras and their downstream proteins in T24 bladder cancer cells. Cells were treated with 200 - 800 μM hyperoside for 48 hours. (a, c) Representative Western blot images of Fas, EGFR, Ras, Akt and MAPKs from three independent experiments with identical results are shown. (b, d) The expression of Fas, EGFR, Ras, Akt and MAPKs was analyzed. Values are mean ± SD. * p < 0.05.

In order to confirm the involvement of Fas and EGFR-Ras signaling pathways in the treatment of hyperoside in human bladder cancer cells, Western blotting was performed to detect the expression levels of Fas, EGFR and Ras, which are upstream proteins in the MAPK and Akt signaling pathways. The results showed that hyperoside significantly increased the expression of Fas, EGFR and Ras at the protein level (Fig 7a and 7b).

Abnormalities in the mitogen-activated protein kinase (MAPK) and the phosphatidylinositol-3-kinase (PI3K)/protein kinase B (Akt) signaling pathways contribute to the carcinogenesis process, including cell proliferation, differentiation, invasion, angiogenesis, apoptosis, and metastasis 12, 13. Thus, the primary subgroups of MAPKs engaged in carcinogenesis, including extracellular signal-regulated kinase (ERK), c-Jun NH2- terminal kinase (JNK) and p38 MAPK, were detected and found to be more phosphorylated in hyperoside-treated T24 cells (Fig 7C-D), which was consistent with the activation of Ras and Fas signaling. Moreover, the expression and phosphorylation levels of Akt were investigated after hyperoside treatment for 24 h. As shown in Fig 7c and 7d, the phosphorylation of Akt at Ser473 was evidently upregulated, which was consistent with Ras overexpression.

Therefore, in contrast to previous studies 9, 10 that hyperoside exerts its anticancer effects through a certain pathway, these phosphorylated MAPK and Akt proteins possess complicated and conflicting downstream signaling pathways, which lead to the ultimate scenario of cell cycle arrest and a small amount of cell apoptosis in T24 bladder cancer cells (Fig. 9).

### Hyperoside suppresses the growth of bladder tumor xenografts in vivo

**Figure 8:**
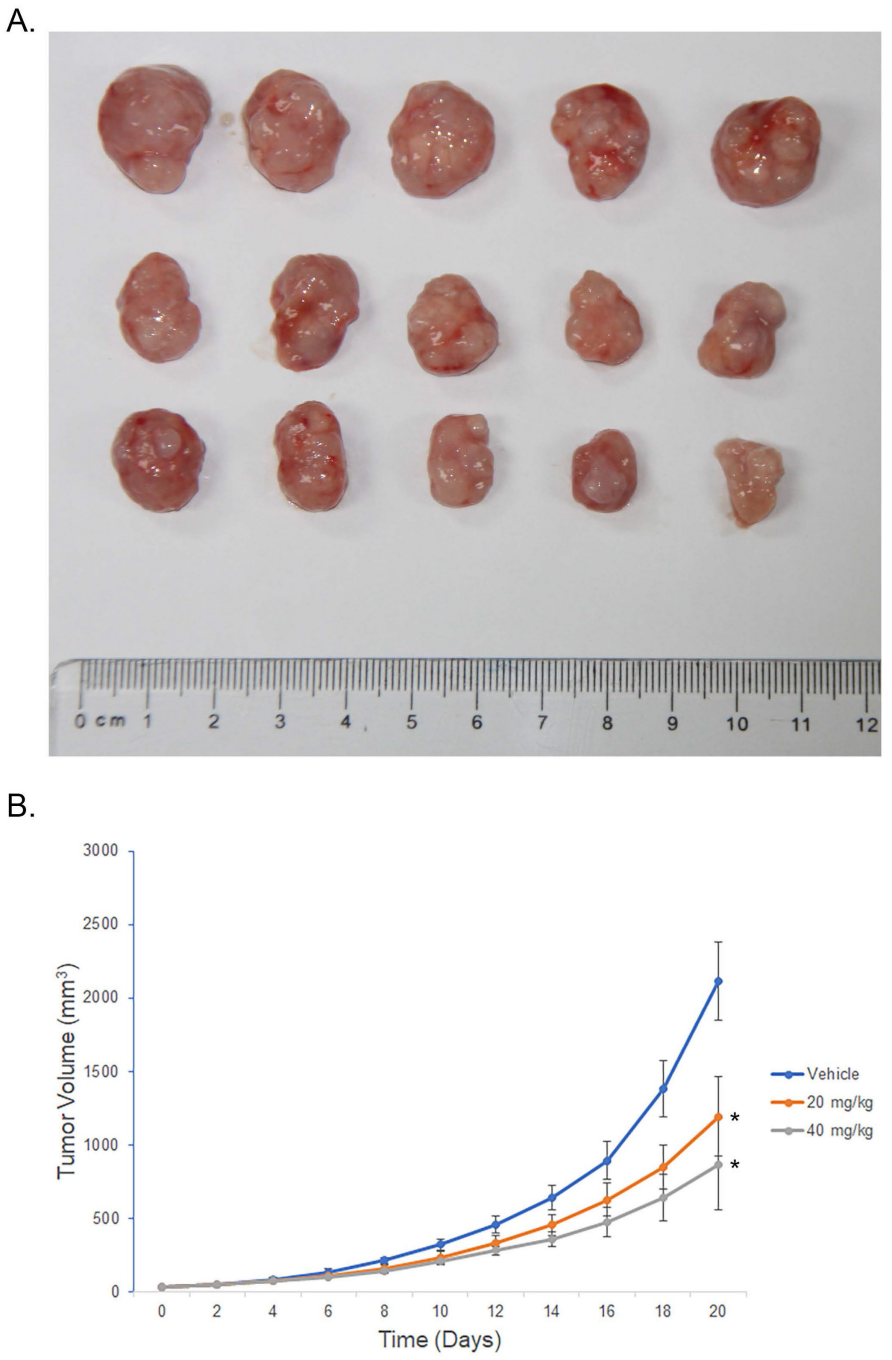
Hyperoside suppresses tumor growth in vivo. Nude mice bearing T24-derived tumor xenografts were injected with hyperoside (20 mg/kg or 40 mg/kg) or vehicle once daily. (a) Representative images of the tumors from three independent experiments with identical results are shown. (b) Tumor curves showed that hyperoside exerted a significant inhibitory effect on the growth of T24-derived tumor xenografts. Values are mean ± SD. * p < 0.05.

**Figure 9:**
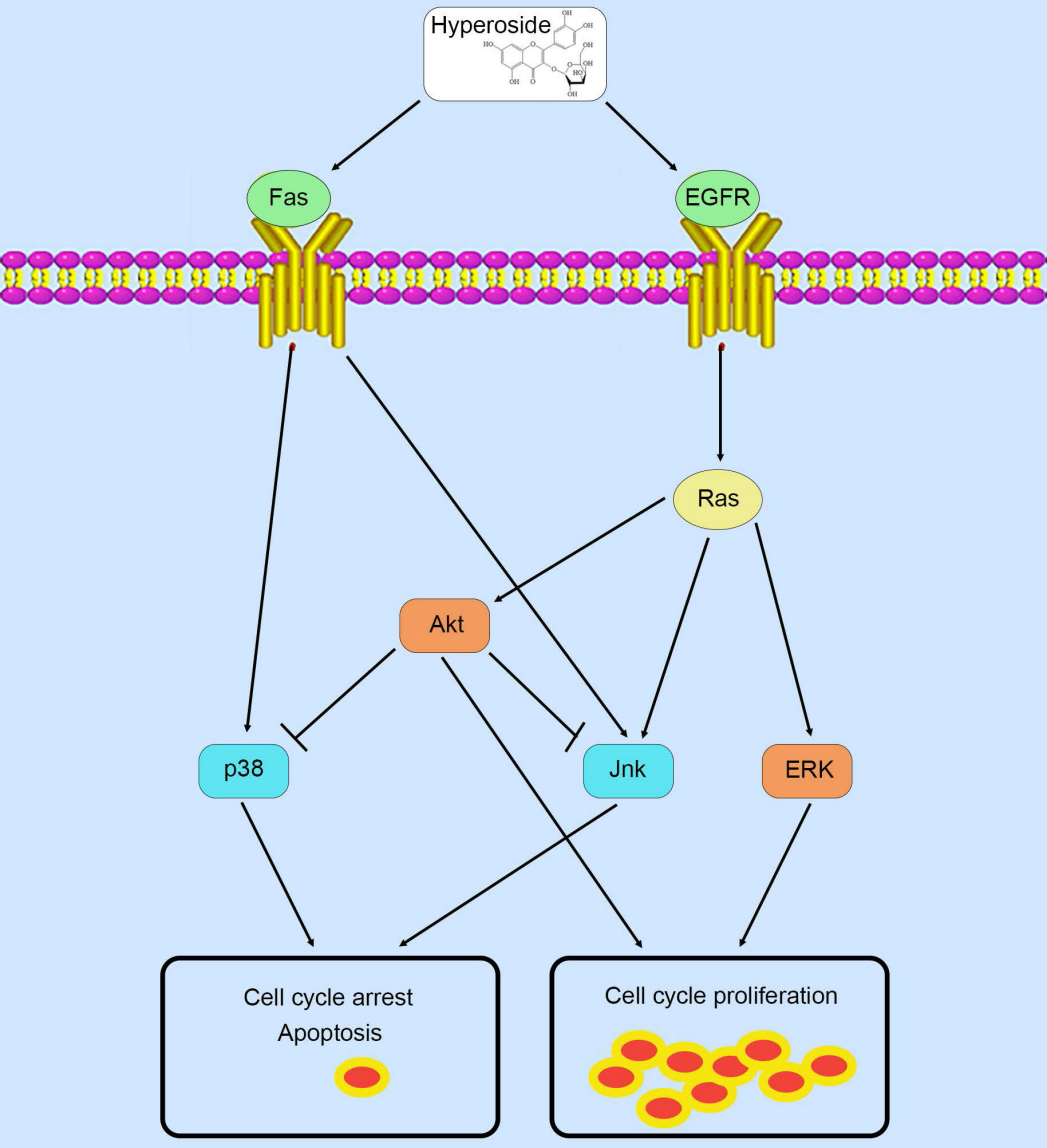
Schematic diagram summarizing the mechanisms of action of hyperoside in bladder cancer cells.

To evaluate the therapeutic potential of hyperoside in cancer treatment, 1×10^5^ T24 cells were subcutaneously injected into the flanks of nude mice. When the tumor size reached about 30 mm^3^, the mice were randomized into three groups and i.p. injected with hyperoside (20 mg/kg and 40 mg/kg) or vehicle control once daily for 3 weeks. As shown in Figure 8, the tumors treated with hyperoside grew more slowly than those in the control group. In the first week, there was no difference in tumor volume between the hyperoside-treated groups and the control group (P > 0.05). In contrast, the tumor volumes of hyperoside-treated groups were significantly smaller than those of the control group from days 11 to 21 (P < 0.05). The results indicated that hyperoside treatment (20 or 40 mg/kg) had antitumor effects on bladder cancer xenografts in vivo.

## Discussion

Hyperoside, also known as quercetin 3-O-beta-D-galactopyranoside, has recently received a lot of attention for its anticancer, anti-inflammatory, antioxidant and organ protective activities. In particular, hyperoside can inhibit cell growth by inducing cell apoptosis and cell cycle arrest in several cancer cells 14-19. However, the exact mechanism of its antitumor effects remains unclear. Some studies attributed this effect to the PI3K/Akt signaling pathway, but others suggested that MAPKs, Fas or NF-κB were also involved in its antitumor activities 9, 10.

Unscheduled proliferation is a hallmark of cancer. Akt and MAPK signaling pathways regulate cell proliferation, survival and differentiation at different transition points, which have a profound impact on the development of various cancers. It is well established that hyperactivation of Akt kinases is a common event in many human cancers, including bladder cancer 20, leading to tumor cell survival and enhanced resistance to apoptosis through multiple mechanisms 21. Overexpression of upstream activators, including epidermal growth factor receptor (EGFR; also known as ERBB1), ERBB2 and/or ERBB3, are associated with the grade, stage and outcome of bladder cancer subsets, and EGFR induces PI3K/Akt activation via RAS activation 22, 23. In our study, hyperoside activated the EGFR-Ras signaling and induced the phosphorylation of Akt at Ser473 in T24 bladder cancer cells, thereby promoting cell proliferation and preventing cell apoptosis.

Unlike Akt, MAPKs have cell type- and context-dependent actions in different cancers, with ERK inhibiting apoptosis and increasing cell proliferation, while JNK and/or p38 are c-Jun/AP-1 and/or p53-mediated apoptotic regulators 24. However, the role of MAPK signaling and its relationship to key mutations in bladder tumors remain unclear. Current data suggest that the majority of bladder cancers may be highly dependent on ERK 25, while both FGFR3 mutations and PIK3CA mutations commonly cooccur in NMIBC, suggesting synergistic activation of MAPK and PI3K pathways 26, 27. Our data showed that all three MAPKs were phosphorylated by hyperoside-induced upstream signals in T24 bladder cancer cells. Taken together, hyperphosphorylated Akt and ERK by the upregulation of EGFR-Ras expression might promote cell proliferation and resist apoptosis, but phosphorylation of JNK and p38 by the activation of Ras or Fas signaling could promote cell cycle arrest and apoptosis conversely.

Previous studies 9, 10 have only focused on and verified the involvement of a single pathway in several cancer cell lines and have not systematically analyzed the role of hyperoside using quantitative proteomics and bioinformatics analysis. In fact, the addition of drug to cells can cause changes in a variety of cellular pathways, eventually resulting in a certain phenotype in a specific cellular and environmental context. In our study, hyperoside induced cell cycle arrest and only a small amount of apoptosis in bladder cancer cells, mainly as a result of the phosphorylation of JNK and p38. The inconspicuous cell apoptosis may be related to the antiapoptotic effects of the phosphorylation of Akt and ERK. Since the hyperactivation of Akt and ERK is already present in most bladder cancers 20, 25, the phosphorylation of Akt and ERK by hyperoside might not significantly enhance their activity further, which could explain why they do not dominate the story of hyperoside.

Although adjuvant therapy can improve treatment outcomes, especially with the great advances in the development of combined therapeutics such as immune checkpoint inhibitors PD-1/PD-L1 inhibitor combined chemotherapy or antibody-drug conjugates (ADCs) or CTLA-4 inhibitors 4, 28, advanced and metastatic bladder cancer can develop resistance to these drugs when treated for a period. Therefore, identification of novel targets and development of effective drugs are urgently needed for bladder cancer treatment. Natural products, especially flavonoids, are an abundant source for screening antitumor drugs due to their remarkable efficacy and low toxicity 6. Our results indicated that hyperoside not only inhibited the proliferation of bladder cancer cells in vitro, but also suppressed the tumorigenesis of bladder cancer in vivo. Well-known cell cycle arrest markers, including cyclins and phosphorylation of CDKs and Rb proteins, were significantly reduced by hyperoside treatment, suggesting that hyperoside might exert its antitumor effects by inducing cell cycle arrest. Moreover, caspase-3 activation plays a central role in apoptosis by cleaving intracellular proteins vital for cell survival and growth, such as PARP 30, leading to the completion of apoptosis and reinforcing the anticancer abilities of hyperoside in bladder cancer cells.

Despite the potent anticancer activity of hyperoside against bladder cancer both in vitro and in vivo, its toxicity and safety should be considered before clinical use. Former studies 9, 10 have already evaluated that 5000 mg/kg hyperoside, more than 600 times the usual dose, only had low acute toxicity in mice without any disorder of their behavior and hematological parameters. Moreover, bacterial reverse mutation assay and chromosome aberration test have been used to indicate that hyperoside has no genetic toxicity. However, long-term and high-dose use of hyperoside was toxic to the kidney and liver of dogs and slowed down the growth of fetal rats, although the damage was negligible and reversible 9, 10. Thus, we can infer that the conventional experimental dose of hyperoside is safe, but more rigorously designed trials are still needed to verify its safety in clinical use in the future.

## Conclusions

Taken together, we revealed for the first time that hyperoside has significant suppressive effects on bladder tumorigenesis in vitro and in vivo by activating EGFR-Ras and Fas signaling pathways. In contrast to previous studies on a single pathway, proteomics and bioinformatics analysis revealed that hyperoside affects multiple signaling pathways, whose synergistic and antagonistic effects ultimately lead to cell cycle arrest in bladder cancer cells under specific contexts and conditions. Regardless, hyperoside offers a new approach to comprehensively prevent bladder cancer. Despite the promise, further studies are needed to screen more natural compounds for cancer treatment and delineate their exact mechanism.

## Abbreviations

μM: μmol/l
IC50: inhibitory concentration 50%
CDK: cyclin-dependent kinase
PARP: poly (ADP-ribose) polymerase
TMT: tandem mass tag
HPLC: high performance liquid chromatography
DEP: differentially expressed protein
PPIs: protein-protein interactions
EGFR: epidermal growth factor receptor
MAPK: mitogen-activated protein kinase
PI3K/Akt: phosphatidylinositol-3-kinase/protein kinase B
ERK: extracellular signal-regulated kinase
JNK: c-Jun NH2-terminal kinase
